# Synthetic Human Lactoferrin Peptide hLF(1-11) Shows Antifungal Activity and Synergism with Fluconazole and Anidulafungin Towards *Candida albicans* and Various Non-Albicans *Candida* Species, Including *Candidozyma auris*

**DOI:** 10.3390/antibiotics14070671

**Published:** 2025-07-02

**Authors:** Carlo Brouwer, Youp van der Linden, Maria Rios Carrasco, Saleh Alwasel, Tarad Abalkhail, Fatimah O. Al-Otibi, Teun Boekhout, Mick M. Welling

**Affiliations:** 1 CBMR Scientific Inc., Edmonton, AB T6J4V9, Canada; c.brouwer@cbmrscientific.com; 2 College of Sciences, King Saud University, Riyadh 11451, Saudi Arabia; salwasel@ksu.edu.sa (S.A.); tabalkhail@ksu.edu.sa (T.A.); falotibi@ksu.edu.sa (F.O.A.-O.); teunboekhout@ksu.edu.sa (T.B.); 3 Westerdijk Fungal Biodiversity Institute, Uppsalalaan 8, 3584 CT Utrecht, The Netherlands; youp1996@hotmail.com (Y.v.d.L.); m.rioscarrasco@uu.nl (M.R.C.); 4 Department of Radiology, Leiden University Medical Center, 2333 ZA Leiden, The Netherlands

**Keywords:** lactoferrin peptide, hLF(1-11), *Candida/Candidozyma auris*, synergy, clinical isolates, drug resistant

## Abstract

Introduction: *Candidozyma auris* (*Cz. auris*) has emerged globally, and diseases caused by it are associated with a mortality rate of 30–72%. This yeast is often multidrug-resistant and challenging to treat. A synthetic peptide, consisting of 11 amino acids of human lactoferrin (hLF1-11), offers a new therapy that is active against *Candida albicans*, non-albicans *Candida* yeasts, as well as *Cz. auris*. The current study examined the susceptibility of clinically relevant *Candida* species to hLF(1-11) in vitro and investigated the synergistic interaction of this peptide with fluconazole (FLU) and anidulafungin (ANI). Methods: Susceptibility of the yeasts to hLF(1-11) was tested with a microdilution method to determine minimum inhibitory concentrations (MICs). A total of 59 strains belonging to 16 species of *Candida* or *Candidozyma* were tested. The treatment cohort included 20 strains of *Cz. auris* originating from six different countries. Results: Mean MIC values of all susceptible strains ranged from 16.66 ± 6.46 μg/mL to 45.83 ± 10.21 μg/mL. There were no statistical differences in the susceptibility of hLF(1-11) for *Cz. auris* across geographic origins. In the combinatory tests, drugs acting together, the fractional inhibitory concentration indexes [FIC] < 1.0, showed a synergistic or additive effect on the efficacy of FLU and ANI when used in combination with hLF(1-11). [FIC] indexes 1–2 were interpreted as intermediate. MIC values in combinatory use were 1–2 titer steps lower than when used alone. Conclusions: hLF(1-11) inhibits the growth of yeasts that belong to the genus *Candida*, including *Cz. auris.* The combinatory use may be further investigated to treat infections caused by resistant yeasts.

## 1. Introduction

Invasive fungal infections (IFIs) are characterized by late and difficult diagnosis and high mortality, affecting an increasing number of immunocompromised patients in hospitals [1]. Due to their public health importance, several of these species are listed on the WHO list of fungal pathogens (accessed on 30 June 2025). *C. albicans*, an opportunistic yeast that can cause mucosal or invasive infections when the host immune system is debilitated, remains the predominant species responsible for candidiasis [2]. However, recent clinical studies have shown an increasing incidence of candidemia due to non-albicans species such as *C. parapsilosis*, *C. tropicalis*, *N. glabratus* (also known as *C. glabrata*), *Pichia kudriavsevii* (also known as *C. krusei*), and, in recent years, *Cz. auris* (also known as *C. auris*) [2,3,4,5,6,7,8].

Many studies have reported the antifungal susceptibility of the clinically most common *Candida* and non-albicans *Candida* species, but limited data exist for the uncommon yeast species. Recently, several studies have investigated the susceptibility of antifungal drugs to several classes of uncommon but emerging yeast species [3,9,10]. Resistance against commonly used antifungal drugs is a well-known phenomenon, whether it is acquired, e.g., *C. albicans* becoming resistant to azoles, or intrinsically present, such as *N. glabratus* showing poor susceptibility to azole and echinocandin drugs, as is likely also the case for several emerging yeast species [2,3,11,12]. Prolonged treatment and prophylactic use strongly increase the likelihood of developing the resistance of various *Candida* species [13]. At present, the antifungal drug arsenal for the treatment of systemic infections is limited to five classes of antifungal drugs, namely the polyenes (amphotericin B), triazoles (e.g., itraconazole and fluconazole) [14], and echinocandins (e.g., caspofungin, micafungin, and anidulafungin) [15]. Flucytosine and allylamine are single classes. Hence, with the increased incidence of drug resistance, there is an urgent need for novel antifungal drugs.

*Candida* (now reclassified as *Candidozyma*) *auris* was described in 2009 based on a single isolate from the external ear canal of an inpatient in Japan [16], and has since then emerged around the globe. More than 10,000 infections with *C. auris* were reported in the USA from 2016 to 2023, and 22,931 people were found to be colonized (https://www.cdc.gov/candida-auris/tracking-c-auris/ (accessed on 27 June 2025)) without showing any symptoms.

*Cz. auris* has been detected in 61 nations spanning six continents. Reports indicate its presence in all subregions of Africa, with over 2500 cases. Significant outbreaks have been noted in specific healthcare environments in South Africa and India, where *Cz. auris* has been linked to as much as 25% and 40% of the candidemia occurrences.

It is argued that these figures are much higher due to a low identification rate [17]. *Cz. auris* mainly colonizes people without giving any symptoms, but an infection with this yeast can lead to various health problems. For example, infections of the ear, wounds, urinary tract, and primarily bloodstream infections are described (European Centre for Disease Prevention and Control. *C. auris* in healthcare settings—Europe—first update, 23 April 2018. Stockholm: ECDC; 2018) [3,15,16,18]. *Cz. auris* infections are related to several risk factors, such as long-term hospitalization, transplantation surgery, invasive procedures, and underlying diseases, such as diabetes or HIV infection. Consequently, ICU patients are most at risk [3,15,17,18,19,20,21]. The mortality rate of a *Cz. auris* infection varies between 30 and 72% [22], depending on the underlying comorbidities of the patients [18].

Treatment of infections with *Cz. auris* is challenging. Symptomatic infections are treated preferably with echinocandins, such as caspofungin, because of the relatively mild side effects [23]. Resistance of *Cz. auris* to commonly used antifungal drugs is caused by a variety of factors, such as mutations in the ERG11 gene (increased copy number of the ERG11 gene), overexpression of efflux pumps such as CDR1 and MDR1, and the excessive and prophylactic use of antifungal agents [3,15,23,24]. Almost all *Cz. auris* isolates are resistant to fluconazole [25]. An increasing problem is the development of drug resistance; *Cz. auris* is recognized for its intrinsic resistance to a broad spectrum of antifungal agents, encompassing those from the azole, echinocandin, and polyene categories. This resistance renders it a challenging pathogen to manage against fluconazole in combination with echinocandins and/or the more toxic polyene amphotericin B [3,17,18,25]. Some isolates of *Cz. auris* are even resistant to all three antifungal antibiotic classes. Thus, *Cz. auris* is an emerging, multidrug-resistant microbe.

In this view, antimicrobial peptides (AMPs) may provide an alternative to the commonly used antifungal treatment regimens for infections caused by *Cz. auris* [3,26,27,28]. Antimicrobial peptides act via different pathways and pose another possibility for reducing the effects of infections [29]. Lactoferrin (LF) is a peptide of 80 kDa that occurs a.o. in the milk and saliva of animals, including humans [30]. Human LF (hLF) has bactericidal activity against a range of pathogens and is suggested to be active against biofilms. A synthetic peptide of human lactoferrin-derived peptide hLF(1-11) (sequence: GRRRRSVQWCA), which has previously been shown to have a broad antimicrobial spectrum, was used in this study [3,28,30,31,32]. The hLF1-11 peptide’s antifungal mechanism involves disrupting fungal cell membranes and targeting mitochondrial function, leading to cell death. It interacts with and enhances immune cell function, fostering a more vigorous antifungal response.

The purpose of the current study was to examine the susceptibility of different, clinically relevant *Candida* species and species that until recently were classified in the genus *Candida*, including *Cz. auris*, to hLF(1-11) in vitro and, secondly, to investigate the interaction of this peptide with two commonly used antifungal agents, fluconazole and anidulafungin for those yeasts.

## 2. Results

### 2.1. Minimum Inhibitory Concentration (MIC) Values

This experiment was conducted to determine the susceptibility of several clinically relevant yeast species that belong to the genus *Candida* or were classified in this genus until recently to hLF(1-11). Emphasis was placed on *Cz. auris*. Fifty-nine strains belonging to 16 different yeast species were evaluated (Table 1).

hL(1-11) was effective against all 59 strains tested, with MIC values ranging from a mean of 25 μg/mL (6.25 μg/mL to 50 μg/mL) in one or two titer steps (Table 1), and all were regarded as susceptible. Negative controls that were incubated solely with cells or incubated with c-LF(1-11) yielded a significantly higher MIC of >100 μg/mL and showed no signs of growth inhibition (Table 2).

Various strains of *Cz. auris* showed MIC values of 12.5 or 25 μg/mL to hLF(1-11) (Table 3).

The range of MIC values of strains belonging to all species tested for FLU and ANI were between 0.25 and 128 μg/mL and between 0.025 and 8 μg/mL, respectively (Table 4). In the combinatory testing of hLF(1-11) with FLU or ANI, the MIC values for both the peptide and the antifungals were lower than when the compounds were tested alone. A comparison of these MIC values can be found in Table 4. The lowest MIC value from the combinatory testing was two dilutions lower (1.56 μg/mL compared to 6.25 μg/mL), and the highest was one dilution lower (25 μg/mL compared to 50 μg/mL) than when hLF(1-11) was tested alone.

### 2.2. Synergy Studies

Seventeen strains of sixteen species were tested in the synergy studies. For FLU, a synergistic effect ([FIC] ≤ 0.5) was present for one strain of *D. rugosa* CBS7138, and for ANI for two strains of *Cz. auris* CBS15279 and *N. nivariensis* CBS 9983. An additive effect ([FIC] = 0.5–1) for FLU was observed for 15 strains belonging to 14 species and for ANI for 13 strains of 12 species. Intermediate [FIC] values ([FIC] = 1–2), which reveal an indifferent effect, were observed for FLU tests against two strains of *C. dubliniensis* CBS 7987 and *Cz. haemuli* CBS 180 and for ANI, three strains: *C. parapsilosis* CBS604, *Cyberlindnera jadinii* CBS 1600, and *Nakaseomyces bracarensis* CBS 10154. An antagonistic effect ([FIC] ≥ 2) was not observed (Table 4).

## 3. Discussion

The main research objective in this study was to evaluate the susceptibility of yeasts that belong to the genus *Candida* or species formerly classified in this genus, emphasizing *Cz. auris* (Table 3). In line with previous studies, a strong antimicrobial effect of hLF(1-11) was found in all strains of all yeast species tested [4,35,41,42]. Furthermore, all the *Cz. auris* group values were either 12.5 μg/mL or 25 μg/mL and were regarded as susceptible. The hLF(1-11) peptide inhibited the growth of *Cz. auris* significantly if compared to a negative control peptide. No correlation was observed between the susceptibility for hLF(1-11) and the geographical origin of the *Cz. auris* strains, but it must be stressed that not all known genetic and geographic diversity was included in this study. Contrary to the strong resistance of *Cz. auris* against currently used antifungal agents, none of the *Cz. auris* strains showed resistance against hLF(1-11). This apparent lack of resistance to hLF(1-11) can be explained by the fact that hLF(1-11) has different working mechanisms than the commonly used antifungal agents. LF is a cationic, basic, and amphipathic peptide that induces Ca^2+^ influx in the mitochondria, leading to a release of adenosine triphosphate (ATP) production and reactive oxygen species (ROS) [41,42,43,44]. Azoles, echinocandins, and polyenes are uncharged molecules interacting with the cell wall and cell membranes; secondly, the yeast has not developed resistance mechanisms, probably because the peptide is not widely used yet [21,38]. It can be argued that it is less likely to develop because LF is present in breast milk and saliva, where it co-exists for longer times with pathogens, including yeasts. Although this study yielded no resistance to hLF(1-11), the literature implies that the long-term resistance to LF development cannot be entirely excluded, but long-term exposure studies are needed to investigate this further [45].

Antimicrobial peptides are ideal candidates for new antifungal therapies as their toxicity is minimal or absent [46], the resistance development rates are low, and they can be used with an antifungal drug. Here, we demonstrated that the synthetic antimicrobial membrane-disruptive and immunomodulatory peptide, consisting of the first amino acids of human antimicrobial lactoferrin peptide (hLF1-11), shows a synergistic or additive effect in the cases of species like *Cz. auris* when used in combination with FLU or ANI. Even for *C. albicans* and *N. glabratus* that is not inherently resistant to antifungals, we noticed, as in former studies, that the combinatory use of hLF(1-11) and the antifungal drug had an additive effect, resulting in a reduced drug dose needed to inhibit the growth of the yeast [6,41,42,47,48]. This additive effect was also true for other species, such as *N. glabratus* and *Cz. auris* that are inherently resistant to azole drugs. Thus, hLF(1-11) might be developed in a drug to combat those yeast species listed as priority fungal pathogens. Importantly, this may result in the decreased development of resistance to commonly used antifungals such as azoles. The results for the potential use of hLF(1-11) in combination with azole drugs and anidulafungin suggest that previously unsuitable antifungal treatment options could be reinstated due to the lower MIC values noted when the antifungal is used in combination with hLF(1-11). Such a treatment can potentially be a solution to treat patients infected with resistant isolates. For instance, *Cz. auris* has developed different kinds of resistance mechanisms. Fluconazole resistance is linked to three different genes. The first mechanism is the efflux of azole drugs through pumps mediated by MDR1/CDR1/CDR2 point mutation [6,11,41,42,49]. Secondly, the gene ERG 11 can be upregulated or mutated. Upregulation of ERG11 results in reduced plasma membrane fluidity and dysfunction of the cell membrane, and a point mutation ensures less binding opportunity between fluconazole and p450 14-α-lanosterol-demethylase [24,41,42,50]. The third mechanism is an ERG5 point mutation, which activates another ergosterol pathway that avoids interaction with azoles [41]. Echinocandin resistance can be caused by a point mutation in FSK1/FSK2 genes, inhibiting the target site [50]. Upcoming resistance against echinocandins and amphotericin B is also associated with biofilm formation, probably the consequence of forming an abiotic surface with phospholipase activity and efflux pumps and a biomechanistic inhibition [51,52]. The utterance of resistance can differ across strains and is associated with the clade they belong to [42,53,54]. Despite differences within a clade, there is a higher amount of genetic variation between clades. For instance, *Cz. auris* strains belonging to the South Asia and South Africa clades have different ERG11 mutations, while no mutation was reported for the strain from Japan (East Asia clade) [20]. Additionally, strains of the East Asia and South Asia (India) clades have a genetic similarity of 63.4% [25,50]. Human LF consists of two similarly sized (α and β) domains, of which the first domain is basic, cationic, and amphipathic [55]. The first 11 amino acids of hLF, i.e., hLF1-11, have the most potent antimicrobial activity as a membrane-disruptive and immunomodulatory peptide [56], and it was known to have a synergistic effect for *C. albicans* in combination with fluconazole therapy [4]. After passing the plasma membrane of yeast pathogens, hLF(1-11) releases Ca^2+^, which is transported to the mitochondria. Eventually, this in transport yields ROS and ATP release, causing a penetrable cell wall and cell death [57]. Additionally, hLF(1-11) enhances the immune system in vivo by stimulating macrophage maturation and activation of the complement system [42,58,59].

Antimicrobial tests using hLF(1-11) have been carried out by comparing logarithmic and stationary yeast stage cells [60]. A study using *C. albicans* supported that log phase cultures gave significant differences to stationary phase cells when adding ketoconazole or miconazole, resulting in a greater distribution of MICs than in the other growth phases [42,61,62]. In our tests with *C. albicans*, no remarkable differences were observed when using logarithmic or stationary phase inocula for this species.

This study showed reliable outcomes because of the low variation found across strains, as the MIC values varied only between 12.5 μg/mL and 25 μg/mL. The variation in the values between these two points can be explained by slight differences among the yeast strains tested or in the hLF(1-11) concentrations used, which may influence the oxidation reaction of AlamarBlue™ in single assays. Previous research has shown that the method of modified MIC tests used in this study is equally reliable compared to standard CLSI guidelines [42,63,64,65]. Another source of variation might be the slightly different growth rates between the isolates, as some strains grew more slowly during incubation in vitro. Slower growth leads to fewer cells and probably also diminished susceptibility [42,66,67].

EUCAST protocols are standardized guidelines used for antifungal testing. The methods recommended include RPMI 1640 [34,42,67], as this showed superior growth of *Candida* spp., regardless of the incubation conditions or the antimicrobial agent used. So far, the best results for antifungal and antibacterial liquid tests have been observed with this medium [42,68,69,70]. In general, MIC values obtained in the RPMI medium in this study were consistent with those obtained in previous studies using the same peptide [34], and the use of this medium has yielded good results for both yeasts and bacteria. Thus, these observations confirm the adequacy of RPMI 1640 for use in our experiments.

Synthetic hLF(1-11) is a safe drug, as shown by safely giving up to several doses of 5 mg/kg in hematopoietic stem cell transplantation patients [46]. Therefore, hLF(1-11) is a promising agent to fight yeast infections, including *Cz. auris*, but it could also play a role in cleaning surfaces and preventing the spread of yeasts. Besides resistance to antifungal agents, the spreading of *Cz. auris* is also a problem for its eradication. Transmission is mainly via contaminated surfaces, as *Cz. auris* attaches to different surfaces and materials [42,71,72]. The complete removal of *Cz. auris* from contaminated surfaces is difficult to treat due to adherence properties and biofilm formation. An infected environment could lead to an outbreak, especially in hospitals. For example, a hospital ward in London (UK) needed to be entirely broken down because the complete eradication of *Cz. auris* appeared to be impossible [73]. Therefore, it is crucial to diagnose and treat infected patients early and search for local sources of yeast. Early detection may also significantly decrease the spread of the disease to other patients.

More extensive studies with increased strains are needed to provide deeper insights into the mechanism of the observed synergistic antifungal effect of hLF(1-11) and azoles or anidulafungin. Also, pharmacodynamic studies are required to test the efficacy of hLF(1-11) alone and in combination with antifungal drugs in animal experiments. Finally, blinded clinical trials are needed to support this concept of synergism between hLF(1-11) and commonly used antifungals in patient and control cohorts. Examples of the use of hLF(1-11) against onychomycosis [74] exist, the data indicate that hLF(1-11) shows no toxicity in humans, and the development of resistance against it seems negligible [75]. The emergence of multidrug-resistant microbial pathogens has created a pressing want for brand new healing options, with LF and its derived peptides, lactoferricins (LFcins), rising as promising applicants because of their multifaceted roles in innate immunity and microbial pathogenesis. LF reveals bacteriostatic and bactericidal properties, promotes immune responses, and has been proven to inhibit viral access and counter microbial mechanisms of infection, making it a precious adjuvant in fighting antibiotic-resistant microorganisms and fungi.

## 4. Materials and Methods

### 4.1. Chemicals

All chemicals used for the experiments described in this manuscript were purchased from commercial sources and used without further purification.

### 4.2. Minimum Inhibitory Concentration (MIC) Values

The MIC of hLF(1-11) against different strains of the yeast species was analyzed using a modified EUCAST broth protocol for susceptibility (method for the determination of broth dilution minimum inhibitory concentrations of antifungal agents for yeasts, EUCAST document E, Def 7.4), (http://www.EUCAST.org, accessed at 30 June 2025) [39,42,76]. The yeast cells were stimulated to grow during incubation of the MIC tests since yeast cells are more susceptible to therapies when in the logarithmic growth phase [77]. AlamarBlueᵀᴹ (Thermo Fisher, Eugene, OR, USA, (https://www.thermofisher.com/order/catalog/product/DAL1025#/DAL1025 (accessed on 27 June 2025)) was used to visualize growth and cell viability [63], The MIC values were determined by measuring the absorbance at 570–600 nm, and the results were compared to equivalent concentrations with an inactive control peptide hLF(1-11) (c-hLF(1-11)) [34].

### 4.3. Candida Strains, Cell Viability Testing

Fifty-nine strains belonging to eight yeast species belonging to Candida or that were recently classified within the genus Candida were obtained from Westerdijk Fungal Biodiversity Institute, Utrecht, The Netherlands. Six strains were obtained from Leiden University Medical Center (LUMC), Leiden, The Netherlands, and three strains from the American Type Culture Collection (ATCC, Rockville, MD, USA) (Table 1). *C. auris* strains from Japan and Korea originated from the East Asian clade, whereas strains from Kuwait, Oman, and India belonged to the South Asian clade. The strains were cultivated for 24–48 h on standard Sabouraud dextrose agar (SDA) plates. Subsequently, a single colony was transferred to a 50 mL sterile bottle containing 25 mL ¼ strength (25%) diluted RPMI 1640 medium (Invitrogen Corp., Thermo Fisher, Walthan, MA) with 10 mM NaPBS and a pH of 7.4, and incubated for 18–20 h (i.e., overnight) by 35 °C under vigorous shaking (150 rpm). From the overnight culture, 3 mL was transferred into a 50 mL sterile bottle containing 25 mL of ¼ strength RPMI 1640 medium. This subculture was pre-warmed (35 °C) and incubated again for 2.5 h under the same conditions to induce growth of the yeast cells. The cells were harvested from the pellet after centrifugation (10 min at 4 °C) at 4000 rpm. This pellet was washed twice with 10 mL of 10 mM NaPBS pH 7.4. Furthermore, the pellet was resuspended in 1 mL of PBS, and the concentrations were determined by reading OD 570–600 nm using a spectrophotometer (SPECTRO star Nano Absorbance Reader, BMG Labtech, Ortenberg, Germany). For the experiments, the concentration of all yeast suspensions was set to 0.5–2.5 × 10^5^ forming units (CFU)/mL [34].

### 4.4. Antimicrobial Peptides

The peptides were purchased from Proteogenix, Schiltigheim, France. The synthetic hLF(1-11) consisted of (Ac-GRRRRSVQWCA-CONH2; MW 141,562 Da; purity > 98%). The negative control (c-hLF(1-11), consisted of the same amini acids as hLF-1-11 with substituted alanines at positions 2, 3, 6, 10 (Ac- GAARRAVQWAA-CONH2; MW 1156.4 Da; purity 96.3%) [34]. The purity of the peptides was determined by reverse-phase high-performance liquid chromatography (HPLC) using a Waters HPLC system with a 1525EF pump and a 2489 UV/VIS detector. For analytical HPLC, a Dr. Maisch GmbH Reprosil-Pur C18-AQ 5 µm (250 × 4.6 mm) or a Dr. Maisch GmbH Reprosil-Pur C18-AQ 5 µm (250 × 10 mm) column was used, with a gradient of 0.1% *v*/*v* trifluoroacetic acid (TFA) in H_2_O/CH_3_CN 95:5 to 0.1% TFA in H_2_O/CH_3_CN 5:95 in 40 min (1 mL/min^−1^). The sample size was 20 mL of a peptide solution of hLF(1-11) (i.e., 1.5 mg/mL water). Stocks of the peptides were diluted at a concentration of 2 mg/mL of 0.01% HAc; pH of 3.7), dried in a Speed-Vac, stored at −20 °C, and thawed immediately before use [26].

### 4.5. EUCAST Broth Microdilution Method

The strains shown in Table 4 were all previously tested according to a modified EUCAST broth protocol for susceptibility to fluconazole (FLU) and anidulafungin (ANI) as described in the Section 3. The two antifungal compounds were purchased from the following companies: fluconazole (FLU), Pfizer Central Research Sandwich, UKITC, Janssen Research Foundation, Beerse, Belgium, and anidulafungin (ANI), Sigma-Aldrich Chemie GmbH, Taufkirchen, Germany.

### 4.6. Minimal Inhibitory Growth Assays

Yeast cultures were tested and treated as described in Section 4.3 above. For the experiments, the concentration of all yeast suspensions was set to 0.5–2.5 × 10^5^ colony forming units (CFUs)/mL [34].

Afterward, concentrations were adjusted to 2 × 10^5^ CFUs/mL into ¼ strength RPMI 1640 medium. Before adding peptides into the microtiter plate (96 wells, u-bottom, low bind, Greiner Bio-one), electrostatic pressure was avoided by placing the plate on top of a wet tissue. The hLF(1-11) working stock solution (2 mg/mL) was prepared as described above. The peptides were pipetted in the microtiter plate in a concentration range of 0–200 μg/mL, supplemented with ¼ strength RPMI 1640 up to a total volume of 100 μL. After adding the peptides, 100 μL of yeast suspensions 2 × 10^5^ CFU/mL dilutions and 1 μL of AlamarBlueᵀᴹ were added to the wells. The negative controls contained only 200 μL ¼ strength RPMI medium or a yeast dilution with AlamarBlueᵀᴹ for growth monitoring. Plates were incubated at 35 °C for 24–28 h under vigorous shaking (120 rpm). Growth was detected when a red/pink color appeared, and inhibition of growth as blue/purple. Color changes by AlamarBlueᵀᴹ were interpreted by the eye and confirmed at OD 570–600 nm with a microplate reader (Bio-Rad, Hercules, CA, USA). MIC values below 100 μg/mL were considered susceptible [27]. The c-LF(1-11) should not show any activity in RPMI media. The substitution of arginine into alanine results in a change in the charge, hydrophobicity, and amphipathic structure of the peptide, which alters the antimicrobial characteristics [10,27,33,78]. As controls, reference strains were incubated for 24–48 h at 35 °C without an agent, and then growth was monitored using a spectrophotometer at OD 600 nm. Results are means of at least five independent experiments (Table 2).

### 4.7. Synergy Studies

**Figure 1:**
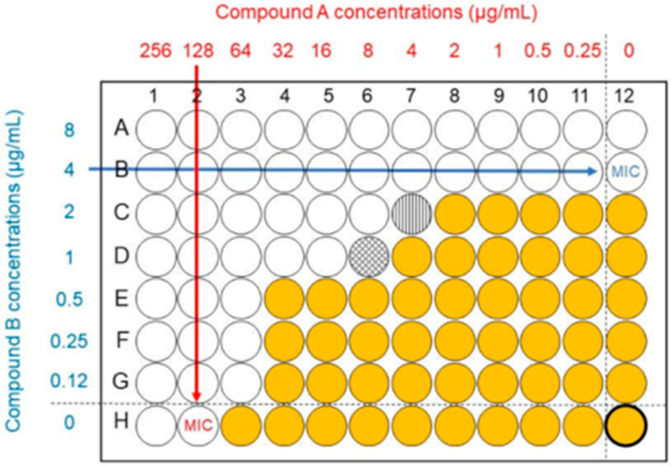
An example of a checkerboard assay is combining two compounds to test increased effectiveness [49].

For the antifungal synergy studies, the cultures were incubated overnight at 35 °C in 25% RPMI 1640 (¼ strength), and the yeast cells were suspended to 2 × 10^5^ CFUs/mL as described above. The hLF(1-11) working stock solution (2 mg/mL) was prepared as described previously. First, hLF(1-11) was dispensed in a 96-well microtiter plate with concentrations ranging from 800 to 0 μg/mL. A horizontal gradient was prepared on the plate with a high concentration on the left side. Secondly, two separate 96-well plates were prepared, one with fluconazole and one with anidulafungin, with concentrations ranging from 512 to 0 and 32 to 0 μg/mL, respectively, in a vertical gradient with the highest concentration at the top side (Figure 1) [79].

Finally, 150 μL of yeast suspension with a concentration of 2 × 10^5^ CFUs/mL was dispensed in all wells of the checkerboard plate, and 25 μL of the peptide was added. Plates were briefly incubated on a shaker at 35 °C for 15 min [34]. Then, 25 μL of the antifungal drug, either fluconazole or anidulafungin, was added to each well. The peptide and the antifungal drug were placed at the same positions as in the original and antifungal drug plates to give a final volume of 200 μL per well [80]. Endpoints were determined by measuring the OD 600 nm at 0, 24, and 48 h after incubating the plates at 35 °C. The plates were agitated before reading to ensure the contents were resuspended. The fractional inhibitory concentration [FIC] index for combinations of two antimicrobials was calculated according to the equation: [FIC] index = [FIC]A + [FIC]B = A/MICA + B/MICB, where A and B are the MICs of drug A and drug B in the combination; MICA and MICB are the MICs of drug A and drug B alone; and [FIC]A and [FIC]B are the [FIC] of drug A and drug B [42]. The [FIC] indexes were interpreted as follows: ≤0.5, synergistic; >0.5–1, additive; 1–2 intermediate; and ≥2.0, antagonistic [34].

## 5. Conclusions

The research on the potential of LF peptides to inhibit *Candida* and similar yeasts, including *Cz. auris*, shows promising results. The LF peptides have strong antifungal properties, making them valuable candidates for developing new treatment options. The small LF peptide hLF(1-11) is considered a promising drug in antifungal interventions but testing its efficacy in animals and eventually in humans is needed. As the prevalence of *Cz. auris* infections continue to rise, and antifungal resistance becomes a growing concern, exploring alternative therapies, such as those using LF-based peptides, could be a significant step forward in addressing this challenge. In the combinatory tests, the fractional inhibitory concentration indexes [FIC] for the tested strains were up to 1.0, showing that there is a synergistic or additive effect on the efficacy of fluconazole and anidulafungin when used in combination with hLF(1-11). MIC values in combinatory use were one or two titer steps lower than when applied alone. The [FIC] indexes of the checkerboard assays were all <1, showing that hLF(1-11) and the antifungal drugs fluconazole or anidulafungin have a synergistic-additive relationship. hLF(1-11) inhibits the growth of *Cz. auris* and other ascomycetous yeasts of clinical relevance and, therefore, has a promising medical application. The combinatory use may have promise in treating infections caused by resistant isolates.

## Figures and Tables

**Table 1:** Minimum inhibitory concentration (MIC) values of the antimicrobial compound hLF(1-11) against selected species of *Candida* or species that were formerly classified in the genus *Candida* [33,34,35,36,37,38,39,40].

Name	Old Name	Strain	Country of Origin	Source	MIC Values	
					Mean	St. Dev
*Candida albicans*		AA (LUMC)	Netherlands	Blood	22.92	5.10
*Candida albicans*		ATCC 90028	United States	Blood	18.75	6.85
*Candida albicans*		ATCC 10231	unknown	Bronchomycosis	23.21	4.73
*Candida albicans*		CBS 562	Uruguay	Skin	20.46	6.31
*Candida albicans*		CMC 1968	Italy	Human	23.21	4.73
*Candida albicans*		Y01-19	unknown	unknown	16.67	6.46
*Candida dubliniensis*		CBS 7987	Iceland	unknown	22.92	5.10
*Candida parapsilosis*		ATCC 22019	United States	Case of sprue	23.44	8.99
*Candida parapsilosis*		CBS 604	Puerto Rico	unknown	20.83	6.46
*Candida parapsilosis*		CMC 2039	Italy	Human	18.75	6.85
*Candida parapsilosis*		LUMC	Netherlands	Human	18.75	6.85
*Candida tropicalis*		CBS 1920	unknown	unknown	21.88	7.66
*Candida tropicalis*		CBS 94	unknown	unknown	45.83	10.21
*Candida tropicalis*		CMC 2041	Italy	Human	41.67	12.91
*Candida tropicalis*		LUMC	Netherlands	Human	23.21	4.73
*Candidozyma auris*	*Candida auris*	2MG-1491	unknown	unknown	21.25	6.04
*Candidozyma auris*	*Candida auris*	CBS 10913	Japan	Ear	21.25	5.88
*Candidozyma auris*	*Candida auris*	CBS 12372	Korea	Blood	19.38	6.38
*Candidozyma auris*	*Candida auris*	CBS 12373	Korea	Blood	21.88	5.55
*Candidozyma auris*	*Candida auris*	CBS 12805	India	Blood	20.00	6.46
*Candidozyma auris*	*Candida auris*	CBS 12806	India	Blood	21.25	6.04
*Candidozyma auris*	*Candida auris*	CBS 12807	India	Blood	20.00	6.46
*Candidozyma auris*	*Candida auris*	CBS 12874	India	Blood	20.00	6.46
*Candidozyma auris*	*Candida auris*	CBS 12875	India	Blood	20.00	6.46
*Candidozyma auris*	*Candida auris*	CBS 12883	India	Blood	20.00	6.46
*Candidozyma auris*	*Candida auris*	CBS 12884	India	Blood	21.25	6.04
*Candidozyma auris*	*Candida auris*	CBS 12885	India	Human	22.50	5.27
*Candidozyma auris*	*Candida auris*	CBS 14144	Kuwait	Blood	18.75	6.59
*Candidozyma auris*	*Candida auris*	CBS 1491	unknown	unknown	20.00	6.85
*Candidozyma auris*	*Candida auris*	CBS 14916	Oman	Blood	18.75	6.59
*Candidozyma auris*	*Candida auris*	CBS 14918	Oman	Blood	20.00	6.46
*Candidozyma auris*	*Candida auris*	CBS 1492	unknown	unknown	20.00	6.85
*Candidozyma auris*	*Candida auris*	CBS 15108	Oman	unknown	20.00	6.46
*Candidozyma auris*	*Candida auris*	CBS 15109	Oman	Human	20.00	6.46
*Candidozyma auris*	*Candida auris*	CBS 15279	Belgium	Kuwaiti patient	18.18	6.53
*Candidozyma duobushaemuli*	*Candida pseudohaemulonii*	CBS 10004	Thailand	unknown	19.79	8.31
*Candidozyma duobushaemuli*	*Candida pseudohaemulonii*	CBS 12371	Korea	unknown	20.83	6.46
*Candidozyma duobushaemuli*	*Candida pseudohaemulonii*	CBS 7798	United States	unknown	22.50	5.59
*Candidozyma duobushaemuli*	*Candida pseudohaemulonii*	CBS 7800	United States	unknown	20.00	6.85
*Candidozyma duobusheamuli*	*Candida duobushaemulonii*	CBS 7798	United States	unknown	21.88	6.25
*Candidozyma duobusheamuli*	*Candida duobushaemulonii*	CBS 7800	United States	unknown	40.00	13.69
*Candidozyma haemuli*	*Candida haemulonii*	CBS 12437	Spain	unknown	22.92	5.10
*Candidozyma haemuli*	*Candida haemulonii*	CBS 12439	Spain	unknown	20.83	6.46
*Candidozyma haemuli*	*Candida intermedia*	CBS 572	Puerto Rico	unknown	22.92	5.10
*Clavispora lusitaniae*	*Candida lusitaniae*	CBS 6936	Israel	unknown	20.83	6.46
*Clavispora lusitaniae*	*Candida lusitaniae*	CMC 1944	Italy	Human	45.83	10.21
*Diutina rugosa*	*Candida rugosa*	CBS 613	unknown	unknown	22.50	5.59
*Diutina rugosa*	*Candida rugosa*	CBS 7138	Netherlands	unknown	22.50	5.59
*Meyerozyma guilliermondii*	*Candida guilliermondii*	CBS 2030	United States	unknown	45.00	11.18
*Nakaeomyces glabratus*	*Candida glabrata*	CBS 138	Italy	unknown	22.73	5.06
*Nakaeomyces glabratus*	*Candida glabrata*	LUMC	Netherlands	Human	22.92	5.10
*Nakaseomyces bracarensis*	*Candida bracarensis*	CBS 10154	Portugal	unknown	21.88	7.66
*Nakaseomyces bracarensis*	*Candida glabrata*	CMC 1933	Italy	Human	23.21	4.73
*Nakaseomyces nivariensis*	*Candida nivariensis*	CBS 9983	Spain	unknown	22.92	5.10
*Pichia inconspicua*	*Candida inconspicua*	CBS 180	Netherlands	unknown	27.08	12.29
*Pichia inconspicua*	*Candida inconspicua*	CBS 1735	Norway	unknown	20.83	6.46
*Pichia kudriavsevii*	*Candida krusei*	LUMC	Netherlands	Human	23.21	4.73
*Pichia kudriavsevii*	*Candida krusei*	CMC 2002	Italy	Human	22.92	5.10
*Pseudolindnera jadinii*	*Candida jadinii*	CBS 1600	France	unknown	22.92	5.10

**Table 2:** Minimum inhibitory concentration values of the negative control peptide, c-hLF(1-11), containing alanine substitutions at positions 2, 3, 6, and 10 against 11 selected yeast strains. Negative controls with only selected strains dilution or c-hLF(1-11) yielded a significantly higher MIC of >100 μg/mL and showed no signs of growth inhibition [34,36,37,38,41]. * = will be soon renamed.

Species	Strain	MIC-Mean
*Candida albicans*	ATCC 90028	>100
*Candida albicans*	ATTC 10231	>100
*Candida albicans*	Y01-19	>100
*Candida parapsilosis* *	ATTC 22019	>100
*Candida parapsilosis* *	LUMC	>100
*Candida tropicalis*	LUMC	>100
*Candidozyma auris*	CBS 10913	>100
*Candidozyma auris*	CBS 12372	>100
*Candidozyma auris*	CBS 15279	>100
*Nakaseomyces glabratus*	LUMC	>100
*Pichia kudriavsevii*	LUMC	>100

**Table 3:** MIC (μg/mL) values of the antimicrobial compound hLF1-11 against 19 strains from five different countries of selected *Candidozyma* strains.

Species	Strain	Country	Mean	St. Dev
			hLF1-11 MIC	
*Cz. auris*	2MG-1491	unknown	21.25	6.04
*Cz. auris*	CBS 10913	Japan	21.25	5.88
*Cz. auris*	CBS 12372	Korea	19.38	6.38
*Cz. auris*	CBS 12373	Korea	21.88	5.55
*Cz. auris*	CBS 12805	India	20.00	6.45
*Cz. auris*	CBS 12806	India	21.25	6.04
*Cz. auris*	CBS 12807	India	20.00	6.45
*Cz. auris*	CBS 12874	India	20.00	6.45
*Cz. auris*	CBS 12875	India	20.00	6.45
*Cz. auris*	CBS 12883	India	20.00	6.45
*Cz. auris*	CBS 12884	India	21.25	6.04
*Cz. auris*	CBS 12885	India	22.50	5.27
*Cz. auris*	CBS 14144	Kuwait	18.75	6.59
*Cz. auris*	CBS 1491	unknown	20.00	6.85
*Cz. auris*	CBS 14916	Oman	18.75	6.59
*Cz. auris*	CBS 14918	Oman	20.00	6.45
*Cz. auris*	CBS 1492	unknown	20.00	6.85
*Cz. auris*	CBS 15108	Oman	20,00	6.45
*Cz. auris*	CBS 15109	Oman	20.00	6.45

**Table 4:** Strains of *Candida* species or those classified in this genus until recently were used for checkerboard combination testing. The MIC values obtained for the fluconazole and anidulafungin with hLF(1-11) from the checkerboard microdilution testing are shown. The [FIC] index for every strain for each antifungal was between 0.5 and 1.0 and 1.0 and 2.0, revealing a synergistic or additive effect between the antifungal drugs and hLF(1-11) [36,37,38,39].

Species	Strain	FLU MIC	ANI MIC	hLF (1-11) MIC	FLU + hLF MIC	ANI + hLF MIC	hLF + FLU MIC	hLF + ANI MIC	FIC hLF + FLU	FIC hLF + ANI
		Eucast			Combination Antifungal + Peptide		Combination Peptide + Antifungal			
*Candida* *albicans*	CBS 562	1	0.25	50	0.5	0.13	12.5	12.5	0.75	0.63
*Candida* *dubliniensis*	CBS 7987	128	0.03	25	64	0.03	12.5	6.25	>1	1
*Candida* *parapsilosis*	CBS 604	2	8	12.5	1	8	3.125	12.5	0.75	>1
*Candida* *tropicalis*	CBS 1920	0.25	0.5	6.25	0.25	0.13	3.13	6.25	0.75	1
*Candidozyma* *auris*	CBS 15279	16	1	25	4	0.25	12.5	3.13	0.75	0.5
*Candidozyma duobushaemuli*	CBS 7800	128	4	50	32	2	12.5	12.5	0.75	0.75
*Candidozyma duobushaemuli*	CBS 12371	64	2	12.5	16	0.25	3.13	6.25	0.75	0.63
*Candidozyma haemuli*	CBS 12439	128	4	12.5	128	0.5	12.5	3.13	>1	0.63
*Candidozyma haemuli*	CBS 180	64	1	12.5	16	0.25	6.25	6.25	1	0.63
*Clavispora* *lusitaniae*	CBS 6936	4	1	12.5	1	0.25	3.13	3.13	0.75	0.63
*Cyberlindnera jadinii*	CBS 1600	8	0.01	12.5	4	0	3.13	6.25	0.75–1.0	>1
*Diotuna* *intermedia*	CBS 572	16	0.5	25	8	0.25	6.25	12.5	0.75	1
*Diutina rugosa*	CBS 7138	16	0.5	25	4	0.25	1.56	6.25	0.5	1
*Meyerozyma guilliermondii*	CBS 2030	32	2	50	16	0.25	3.13	3.13	0.75	0.63
*Nakaeomyces glabratus*	CBS 138	8	0.25	12.5	2	2	6.25	6.25	0.75	1
*Nakaseomyces bracarensis*	CBS 10154	4	8	6.25	1	4	3.125	6.25	0.75	>1
*Nakaseomyces nivariensis*	CBS 9983	16	1	12.5	4	0.05	6.25	3.13	0.75	0.5
*Pichia* *kudriavsevii*	CMC 2002	32	0.06	25	8	0.03	12.5	12.5	0.75	1

## Data Availability

Data are contained within the article.

## References

[1] Ellis M. (2002). Invasive fungal infections: Evolving challenges for diagnosis and therapeutics. Mol. Immunol..

[2] Stavrou A.A., Lackner M., Lass-Flörl C., Boekhout T. (2019). The changing spectrum of Saccharomycotina yeasts causing candidemia: Phylogeny mirrors antifungal susceptibility patterns for azole drugs and amphothericin B. FEMS Yeast Res..

[3] Masson P.L., Heremans J.F., Prignot J.J., Wauters G. (1966). Immunohistochemical localization and bacteriostatic properties of an iron-binding protein from bronchial mucus. Thorax.

[4] Lupetti A., Nibbering P.H., Campa M., Del Tacca M., Danesi R. (2003). Molecular targeted treatments for fungal infections: The role of drug combinations. Trends Mol. Med..

[5] Lum K.Y., Tay S.T., Le C.F., Lee V.S., Sabri N.H., Velayuthan R.D., Hassan H., Sekaran S.D. (2015). Activity of Novel Synthetic Peptides against *Candida albicans*. Sci. Rep..

[6] Corrêa-Moreira D., Baptista B.O., Giosa D., Oliveira M.M.E. (2024). Editorial: Emerging fungal pathogens: Perspectives. Front. Fungal Biol..

[7] Pfaller M.A., Andes D.R., Diekema D.J., Horn D.L., Reboli A.C., Rotstein C., Franks B., Azie N.E. (2014). Epidemiology and outcomes of invasive candidiasis due to non-albicans species of Candida in 2496 patients: Data from the Prospective Antifungal Therapy (PATH) registry 2004–2008. PLoS ONE.

[8] Morales-López S.E., Parra-Giraldo C.M., Ceballos-Garzón A., Martínez H.P., Rodríguez G.J., Álvarez-Moreno C.A., Rodríguez J.Y. (2017). Invasive Infections with Multidrug-Resistant Yeast Candida auris, Colombia. Emerg. Infect. Dis..

[9] Pérez-Hansen A., Lass-Flörl C., Lackner M. (2019). Antifungal susceptibility profiles of rare ascomycetous yeasts. J. Antimicrob. Chemother..

[10] Stavrou A.A., Pérez-Hansen A., Lackner M., Lass-Flörl C., Boekhout T. (2020). Elevated minimum inhibitory concentrations to antifungal drugs prevail in 14 rare species of candidemia-causing Saccharomycotina yeasts. Med. Mycol..

[11] Pristov K.E., Ghannoum M.A. (2019). Resistance of Candida to azoles and echinocandins worldwide. Clin. Microbiol. Infect..

[12] Lass-Flörl C., Kanj S.S., Govender N.P., Thompson G.R., Ostrosky-Zeichner L., Govrins M.A. (2024). Invasive candidiasis. Nat. Rev. Dis. Primers.

[13] Arendrup M.C., Patterson T.F. (2017). Multidrug-Resistant Candida: Epidemiology, Molecular Mechanisms, and Treatment. J. Infect. Dis..

[14] Peyton L.R., Gallagher S., Hashemzadeh M. (2015). Triazole antifungals: A review. Drugs Today.

[15] Ghannoum M.A., Rice L.B. (1999). Antifungal agents: Mode of action, mechanisms of resistance, and correlation of these mechanisms with bacterial resistance. Clin. Microbiol. Rev..

[16] Satoh K., Makimura K., Hasumi Y., Nishiyama Y., Uchida K., Yamaguchi H. (2009). *Candida auris* sp. nov., a novel ascomycetous yeast isolated from the external ear canal of an inpatient in a Japanese hospital. Microbiol. Immunol..

[17] Ademe M., Girma F. (2020). *Candida auris*: From Multidrug Resistance to Pan-Resistant Strains. Infect. Drug. Resist..

[18] Lee W.G., Shin J.H., Uh Y., Kang M.G., Kim S.H., Park K.H., Jang H.C. (2011). First three reported cases of nosocomial fungemia caused by *Candida auris*. J. Clin. Microbiol..

[19] Adams E., Quinn M., Tsay S., Poirot E., Chaturvedi S., Southwick K., Greenko J., Fernandez R., Kallen A., Vallabhaneni S. (2018). *Candida auris* in Healthcare Facilities, New York, USA, 2013–2017. Emerg. Infect. Dis..

[20] Lockhart S.R., Etienne K.A., Vallabhaneni S., Farooqi J., Chowdhary A., Govender N.P., Colombo A.L., Calvo B., Cuomo C.A., Desjardins C.A. (2017). Simultaneous Emergence of Multidrug-Resistant *Candida auris* on 3 Continents Confirmed by Whole-Genome Sequencing and Epidemiological Analyses. Clin. Infect. Dis..

[21] Lee J.S., Im D.S., An Y.-S., Hong J.M., Gwag B.J., Joo I.S. (2011). Chronic cerebral hypoperfusion in a mouse model of Alzheimer’s disease: An additional contributing factor of cognitive impairment. Neurosci. Lett..

[22] Forsberg K., Woodworth K., Walters M., Berkow E.L., Jackson B., Chiller T., Vallabhaneni S. (2019). *Candida auris*: The recent emergence of a multidrug-resistant fungal pathogen. Med. Mycol..

[23] Sanglard D. (2016). Emerging Threats in Antifungal-Resistant Fungal Pathogens. Front. Med..

[24] Perlin D.S., Shor E., Zhao Y. (2015). Update on Antifungal Drug Resistance. Curr. Clin. Microbiol. Rep..

[25] Chowdhary A., Sharma C., Duggal S., Agarwal K., Prakash A., Singh P.K., Jain S., Kathuria S., Randhawa H.S., Hagen F. (2013). New clonal strain of *Candida auris*, Delhi, India. Emerg. Infect. Dis..

[26] Zhang Q., Choi K., Wang X., Xi L., Lu S. (2025). The Contribution of Human Antimicrobial Peptides to Fungi. Int. J. Mol. Sci..

[27] Brouwer C., Theelen B., van der Linden Y., Sarink N., Rahman M., Alwasel S., Cafarchia C., Welling M.M., Boekhout T. (2024). Combinatory Use of hLF(1-11), a Synthetic Peptide Derived from Human Lactoferrin, and Fluconazole/Amphotericin B against *Malassezia furfur* Reveals a Synergistic/Additive Antifungal Effect. Antibiotics.

[28] Brouwer C., Welling M.M., Alwasel S., Boekhout T. (2025). Potential health benefits of lactoferrin and derived peptides—How to qualify as a medical device?. Crit. Rev. Microbiol..

[29] Mba I.E., Nweze E.I. (2022). Antimicrobial Peptides Therapy: An Emerging Alternative for Treating Drug-Resistant Bacteria. Yale J. Biol. Med..

[30] Cezara B., Corina C. (2024). Antimicrobial peptides: Opportunities and challenges in overcoming resistance. Microbiol. Res..

[31] Ammons M.C., Copié V. (2013). Mini-review: Lactoferrin: A bioinspired, anti-biofilm therapeutic. Biofouling.

[32] Masson P.L., Heremans J.F., Ferin J. (1968). Presence of an Iron-binding protein (lactoferrin) in the genital tract of the human female. I. Its immunohistochemical localization in the endometrium. Fertil. Steril..

[33] Brouwer C., Boekhout T., Alwasel S., Rahman M., Janga R., Welling M.M. (2024). Screening sensibility and antifungal activity after topical application of a synthetic lactoferrin-derived antimicrobial peptide. Am. J. Transl. Res..

[34] Brouwer C., Roscini L., Cardinali G., Corte L., Pierantoni D.C., Robert V., Rahman M., Welling M.M. (2018). Structure-activity relationship study of synthetic variants derived from the highly potent human antimicrobial peptide hLF (1-11). Cohesive J. Microbiol. Infect. Dis..

[35] Lupetti A., Brouwer C., Bogaards S.J.P., Welling M.M., de Heer E., Campa M., van Dissel J.T., Friesen R.H.E., Nibbering P.H. (2007). Human lactoferrin-derived peptide’s antifungal activities against disseminated *Candida albicans* infection. J. Infect. Dis..

[36] Kurtzman C., Fell J., Boekhout T. (2011). Definition, classification and nomenclature of the yeasts. The Yeasts: A Taxonomic Study.

[37] Li W., Liu B., Lin Y., Xue P., Lu Y., Song S., Li Y., Szeto I.M., Ren F., Guo H. (2024). The application of lactoferrin in infant formula: The past, present and future. Crit. Rev. Food Sci. Nutr..

[38] Takashima M., Sugita T. (2022). Taxonomy of Pathogenic Yeasts *Candida*, *Cryptococcus*, *Malassezia*, and *Trichosporon*. Med. Mycol. J..

[39] Khunnamwong P., Lertwattanasakul N., Jindamorakot S., Limtong S., Lachance M.A. (2015). Description of *Diutina* gen. nov., *Diutina siamensis*, f.a. sp. nov., and reassignment of *Candida catenulata*, *Candida mesorugosa*, *Candida neorugosa*, *Candida pseudorugosa*, *Candida ranongensis*, *Candida rugosa* and *Candida scorzettiae* to the genus *Diutina*. Int. J. Syst. Evol. Microbiol..

[40] Zhu H.Y., Guo L.C., Hu S., Wei Y.H., Hui F.L., Liu X.Z., Bai F.Y. (2024). *Pichia kurtzmaniana* f.a. sp. nov., with the transfer of eight *Candida* species to Pichia. Int. J. Syst. Evol. Microbiol..

[41] Warshaw E.M., Fett D.D., Bloomfield H.E., Grill J.P., Nelson D.B., Quintero V., Carver S.M., Zielke G.R., Lederle F.A. (2005). Pulse versus continuous terbinafine for onychomycosis: A randomized, double-blind, controlled trial. J. Am. Acad. Dermatol..

[42] Thappeta K.R.V., Vikhe Y.S., Yong A.M.H., Chan-Park M.B., Kline K.A. (2020). Combined Efficacy of an Antimicrobial Cationic Peptide Polymer with Conventional Antibiotics to Combat Multidrug-Resistant Pathogens. ACS Infect. Dis..

[43] Brouwer C.P., Rahman M., Welling M.M. (2011). Discovery and development of a synthetic peptide derived from lactoferrin for clinical use. Peptides.

[44] Lupetti A., Pauwels E.K.J., Nibbering P.H., Weling M.M. (2003). Tc-99m-antimicrobial peptides: Promising candidates for infection imaging. Q. J. Nucl. Med..

[45] Zasloff M. (2002). Antimicrobial peptides of multicellular organisms. Nature.

[46] van der Velden W., van Iersel T.M.P., Blijlevens N.M.A., Donnelly J.P. (2009). Safety and tolerability of the antimicrobial peptide human lactoferrin 1-11 (hLF1-11). BMC Med..

[47] Beardsley J., Kim H.Y., Dao A., Kidd S., Alastruey-Izquierdo A., Sorrell T.C., Tacconelli E., Chakrabarti A., Harrison T.S., Bongomin F. (2024). *Candida glabrata* (*Nakaseomyces glabrata*): A systematic review of clinical and microbiological data from 2011 to 2021 to inform the World Health Organization Fungal Priority Pathogens List. Med. Mycol..

[48] Fisher M.C., Denning D.W. (2023). The WHO fungal priority pathogens list as a game-changer. Nat. Rev. Microbiol..

[49] Rybak J.M., Doorley L.A., Nishimoto A.T., Barker K.S., Palmer G.E., Rogers P.D. (2019). Abrogation of Triazole Resistance upon Deletion of CDR1 in a Clinical Isolate of *Candida auris*. Antimicrob. Agents Chemother..

[50] Chowdhary A., Prakash A., Sharma C., Kordalewska M., Kumar A., Sarma S., Tarai B., Singh A., Upadhyaya G., Upadhyay S. (2018). A multicentre study of antifungal susceptibility patterns among 350 *Candida auris* isolates (2009–17) in India: Role of the ERG11 and FKS1 genes in azole and echinocandin resistance. J. Antimicrob. Chemother..

[51] Sherry L., Rajendran R., Lappin D.F., Borghi E., Perdoni F., Falleni M., Tosi D., Smith K., Williams C., Jones B. (2014). Biofilms formed by *Candida albicans* bloodstream isolates display phenotypic and transcriptional heterogeneity that are associated with resistance and pathogenicity. BMC Microbiol..

[52] Sherry L., Ramage G., Kean R., Borman A., Johnson E.M., Richardson M.D., Rautemaa-Richardson R. (2017). Biofilm-Forming Capability of Highly Virulent, Multidrug-Resistant *Candida auris*. Emerg. Infect. Dis..

[53] Czajka K.M., Venkataraman K., Brabant-Kirwan D., Santi S.A., Verschoor C., Appanna V.D., Singh R., Saunders D.P., Tharmalingam S. (2023). Molecular Mechanisms Associated with Antifungal Resistance in Pathogenic *Candida* Species. Cells.

[54] Windsor R.E., Insall J.N., Urs W.K., Miller D.V., Brause B.D. (1990). Two-stage reimplantation for the salvage of total knee arthroplasty complicated by infection. Further follow-up and refinement of indications. JBJS.

[55] Bluard-Deconinck J.M., Williams J., Evans R.W., van Snick J., Osinski P.A., Masson P.L. (1978). Iron-binding fragments from the N-terminal and C-terminal regions of human lactoferrin. Biochem. J..

[56] Nibbering P.H., Ravensbergen E., Welling M.M., van Berkel L.A., van Berkel P.H.C., Pauwels E.K.J., Nuijens J.H. (2001). Human lactoferrin and peptides derived from its N terminus are highly effective against infections with antibiotic-resistant bacteria. Infect. Immun..

[57] Lupetti A., Paulusma-Annema A., Welling M.M., Dogterom-Ballering H., Brouwer C.P.J.M., Senesi S., Dissel J.T.v., Nibbering P.H. (2003). Synergistic Activity of the N-Terminal Peptide of Human Lactoferrin and Fluconazole against *Candida* Species. Antimicrob. Agents Chemother..

[58] van der Does A.M., Bogaards S.J.P., Ravensbergen B., Beekhuizen H., van Dissel J.T., Nibbering P.H. (2010). Antimicrobial peptide hLF1-11 directs granulocyte-macrophage colony-stimulating factor-driven monocyte differentiation toward macrophages with enhanced recognition and clearance of pathogens. Antimicrob. Agents Chemother..

[59] van der Does A.M., Joosten S.a., Vroomans E., Bogaards S.J.P., van Meijgaarden K.E., Ottenhoff T.H.M., van Dissel J.T., Nibbering P.H. (2012). The antimicrobial peptide hLF1-11 drives monocyte-dendritic cell differentiation toward dendritic cells that promote antifungal responses and enhance Th17 polarization. J. Innate Immun..

[60] Trilles L., Fernández-Torres B., Dos Santos Lazéra M., Wanke B., de Oliveira Schubach A., de Almeida Paes R., Inza I., Guarro J. (2005). In vitro antifungal susceptibilities of *Sporothrix schenckii* in two growth phases. Antimicrob. Agents Chemother..

[61] Beggs W.H. (1984). Growth phase in relation to ketoconazole and miconazole susceptibilities of *Candida albican*s. Antimicrob. Agents Chemother..

[62] Meletiadis J., Meis J.F., Mouton J.W., Verweij P.E. (2001). Analysis of growth characteristics of filamentous fungi in different nutrient media. J. Clin. Microbiol..

[63] Cudic M., Condie B.A., Weiner D.J., Lysenko E.S., Xiang Z.Q., Insug O., Bulet P., Otvos L. (2002). Development of novel antibacterial peptides that kill resistant isolates. Peptides.

[64] Cudic M., Lockatell C.V., Johnson D.E., Otvos L. (2003). In vitro and in vivo activity of an antibacterial peptide analog against uropathogens. Peptides.

[65] To W.K., Fothergill A.W., Rinaldi M.G. (1995). Comparative evaluation of macrodilution and alamar colorimetric microdilution broth methods for antifungal susceptibility testing of yeast isolates. J. Clin. Microbiol..

[66] Svensäter G., Björnsson O., Hamilton I.R. (2001). Effect of carbon starvation and proteolytic activity on stationary-phase acid tolerance of *Streptococcus mutans*. Microbiology.

[67] Radetsky M., Wheeler R.C., Roe M.H., Todd J.K. (1986). Microtiter broth dilution method for yeast susceptibility testing with validation by clinical outcome. J. Clin. Microbiol..

[68] Pfaller M.A., Rinaldi M.G., Galgiani J.N., Bartlett M.S., Body B.A., Espinel-Ingroff A., Fromtling R.A., Hall G.S., Hughes C.E., Odds F.C. (1990). Collaborative investigation of variables in susceptibility testing of yeasts. Antimicrob. Agents Chemother..

[69] Sheehan D.J., Espinel-Ingroff A., Moore L.S., Webb C.D. (1993). Antifungal susceptibility testing of yeasts: A brief overview. Clin. Infect. Dis..

[70] Rodríguez-Tudela J.L., Berenguer J., Martínez-Suárez J.V., Sanchez R. (1996). Comparison of a spectrophotometric microdilution method with RPMI-2% glucose with the National Committee for Clinical Laboratory Standards reference macrodilution method M27-P for in vitro susceptibility testing of amphotericin B, flucytosine, and fluconazole against *Candida albicans*. Antimicrob. Agents Chemother..

[71] Kean R., Delaney C., Rajendran R., Sherry L., Metcalfe R., Thomas R., McLean W., Williams C., Ramage G. (2018). Gaining Insights from Candida Biofilm Heterogeneity: One Size Does Not Fit All. J. Fungi.

[72] Piedrahita C.T., Cadnum J.L., Jencson A.L., Shaikh A.A., Ghannoum M.A., Donskey C.J. (2017). Environmental Surfaces in Healthcare Facilities are a Potential Source for Transmission of *Candida auris* and Other Candida Species. Infect Control Hosp. Epidemiol..

[73] Schelenz S., Hagen F., Rhodes J.L., Abdolrasouli A., Chowdhary A., Hall A., Ryan L., Shackleton J., Trimlett R., Meis J.F. (2016). First hospital outbreak of the globally emerging *Candida auris* in a European hospital. Antimicrob. Resist. Infect. Control.

[74] Mercer D.K., Torres M.D.T., Duay S.S., Lovie E., Simpson L., von Köckritz-Blickwede M., de la Fuente-Nunez C., O’Neil D.A., Angeles-Boza A.M. (2020). Antimicrobial Susceptibility Testing of Antimicrobial Peptides to Better Predict Efficacy. Front. Cell Infect. Microbiol..

[75] Vaidyanathan G., Zalutsky M.R. (2011). Applications of 211At and 223Ra in targeted alpha-particle radiotherapy. Curr. Radiopharm..

[76] Buda De Cesare G., Cristy S.A., Garsin D.A., Lorenz M.C. (2020). Antimicrobial Peptides: A New Frontier in Antifungal Therapy. mBio.

[77] McLeod G.I., Spector M.P. (1996). Starvation- and Stationary-phase-induced resistance to the antimicrobial peptide polymyxin B in *Salmonella typhimurium* is RpoS (sigma(S)) independent and occurs through both phoP-dependent and -independent pathways. J. Bacteriol..

[78] Vctor L. (2005). Antibiotics in Laboratory Medicine.

[79] Woods R.J., Read A.F. (2023). Combination antimicrobial therapy to manage resistance. Evol. Med. Public Health.

[80] Thayna A.M.S., Erica O.M., Gabriel B.T., Felipe F.M., Sergio Henrique S., André O.C., Valdirene M.G. (2024). Synergistic action of synthetic peptides and amphotericin B causes disruption of the plasma membrane and cell wall in *Candida albicans*. Biosci. Rep..

